# Carotid Thrombosis in a Crack Cocaine Smoker Woman

**DOI:** 10.1155/2020/4894825

**Published:** 2020-10-06

**Authors:** Mattia Cosenza, Luigi Panza, Anna Paola Califano, Carolina Defendini, Maria D'Andria, Roberto Romiti, Antonio Fabio Massimo Vainieri, Sergio Morelli

**Affiliations:** Department of Internal Medicine and Atherosclerosis Prevention, University of Rome, La Sapienza, Italy

## Abstract

**Introduction:**

We report a case of stroke in a crack smoker with occlusion of the middle cerebral artery and a large thrombus in the carotid artery. *Case Presentation*. A 34-year-old female presented with left upper arm weakness, associated with paresthesia with onset of symptoms more than 24 hours before. Angio-RM sequences showed an area of ischemia, with occlusion of the M2 segment of the middle cerebral artery. Carotid ultrasound showed a soft plaque with distal end floating. Anticoagulant treatment was started, and seriated ultrasound evaluations showed its gradual dissolution.

**Conclusions:**

In atherothromboembolic stroke from carotid thrombosis, repeated ultrasound studies may be useful for either diagnosis and monitoring the efficacy of anticoagulant therapy.

## 1. Introduction

Cocaine use may be associated with stroke and carotid thrombosis. In the emergency setting, this abuse must be suspected in case of stroke of young adults. Accelerated atherosclerosis of carotid arteries can lead to thrombus formation. Whether the medical or surgical approach is to be preferred, it is unclear.

## 2. Case Presentation

A 34-years-old Caucasian female presented with left upper arm weakness associated with paresthesia. The symptoms had started about 24 hours before, on waking.

Physical examination revealed left hemiparesis, with diminished left upper (2/5) and lower (3/5) strength, and extensor plantar response on the left side. There were no cerebellar signs, and the cranial nerve examination was normal. The electrocardiogram showed normal sinus rhythm at 70 beats per minute, and blood pressure values were 160/90 mmHg. No other remarkable signs were present.

Cranial multimodal computed tomography (CT) imaging was performed, showing no sign of cerebral hemorrhage and hypodensity on cortical and subcortical right frontoparietal regions, compatible with ischemic stroke. Thrombolysis was not performed because of the remote onset of the symptoms. Cranial Magnetic Resonance Imaging (MRI) showed an area of ischemia in the acute/subacute phase, without hemorrhagic infarction, with occlusion of the M2 segment of the middle cerebral artery. She was given a loading dose of acetylsalicylic acid and transferred to our division.

Collecting a complete medical history, the patient revealed to be a habitual consumer of substances like heroine, methadone, cocaine, and alcohol; moreover, in the night before the episode, she had smoked crack cocaine. A toxicological screen resulted positive for cocaine and opioids. ECG and cardiac ultrasound were normal.

**Figure 1:**
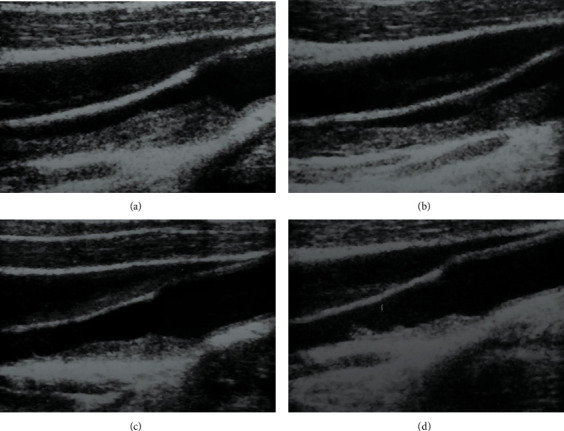
(a, b) A huge homogeneous soft plaque with irregular surfaces and circumferential blood flow at the distal end along carotid bifurcation. (c) Image at 1 week after starting therapy. (d) Image at 2 weeks after anticoagulant.

Carotid ultrasound (CU) showed a huge homogeneous soft plaque with an irregular surface, protrusive morphology, and circumferential blood flow at the distal end, inside the internal carotid artery (Figure 1).

Enoxaparin at the dosage of 100 U/kg twice/day was started, followed by warfarin therapy. Seriated CUs showed gradual dissolution of the thrombus (Figure 1). Her symptoms gradually improved during the next weeks, and she was discharged from the hospital in good general conditions. However, the patient continued drug abusing, and a new cerebral stroke occurred the year after.

## 3. Discussion

Stroke is a note complication of cocaine abuse [1]. The main peak in the description of cocaine-associated stroke happened in ‘80s-‘90s, when crack use began. Crack cocaine has been associated with both ischemic and hemorrhagic stroke, whereas cocaine hydrochloride results more often in hemorrhagic events [2]. Recently, smoked cocaine intake was associated with stroke within 24 hours [3]. Ischemic infarctions usually affect the territories of midcerebral artery [4].

Many mechanisms may explain cocaine and crack cocaine neurotoxicity: vasospasm, endothelial damage, platelet dysfunction, and intracranial vasculitis [5]. When large vessels are involved, mechanisms include accelerated atherosclerosis with large lipid core [6]. Some authors suggest the possibility of vasospasm of large arteries and secondary intravascular thrombosis [7].

Free-floating thrombus is an uncommon condition, defined as an elongated thrombus attached to an arterial wall, with circumferential blood-flow at its distal end and cyclical motion relating to the cardiac cycle, with atherosclerosis as the most common etiology [8]. Both medical and surgical approaches were used, without clear evidence of the superiority of one over the other. In the last years, endovascular procedures were reported [9]. Nevertheless, medical therapy alone showed good results, and complete resolution of thrombosis was described [10–12].

To our knowledge, our case is the first that described the resolution of an intracarotid thrombus related to the assumption of crack-cocaine with medical therapy alone. The population of cocaine smokers is at high risk of stroke, and the management of these patients is complicated by low compliance and low adherence to therapy. The finding of a free-floating thrombus inside a supraaortic vessel is a hard clinical issue to face. The management of these patients is still uncertain, and more studies are required in this regard. Carotid ultrasound allowed to manage this patient-guiding therapy and follow-up.

## Data Availability

No other datas available.
